# Supplementary material for: SPMLMI: predicting lncRNA–miRNA interactions in humans using a structural perturbation method
Source: PeerJ. 2021 May 19;9:e11426. doi: 10.7717/peerj.11426 (PMC8140594; doi:10.7717/peerj.11426)
Supplement: Supplemental Information 1 [file peerj-09-11426-s001.pdf]

Supplementary_File_S1

1 Structural consistency

The concept of structural consistency was first proposed by Lü et al., which can be used to quantify the link predictability of complex networks (Lü et al. 2015; Zeng et al. 2018). They define it as the consistency of network structure features before and after random removal of partial links. In this study, we applied this method to weighted bilayer networks; viz. weighted lncRNA–miRNA bilayer network A.

We use graph $G=(V,E,W)$ to represent the weighted lncRNA–miRNA bilayer network. V and E are the sets of nodes (include both lncRNA and miRNA nodes) and edges, respectively; and W is set of weights. We select a small part of the links to compose a perturbation set ΔE, while the rest of the links are defined as E^R. ΔA and A^R represent the corresponding weighted adjacency matrix, respectively; and $A=A^R+\Delta A$. Obviously, A^R is a real symmetric matrix; therefore, it can be diagonalized as follows.

$$A^R=\sum_{k=1}^{N}\lambda_k x_k x_k^T , \tag{2}$$

where λ_k are the eigenvalues for A^R and x_k are the corresponding orthogonal and normalized eigenvectors.

Using ΔE as the perturbation set, we obtain a perturbed matrix by first-order approximation. First-order approximation allows the eigenvalues to change but keeps the eigenvectors constant. Two cases are considered. First, consider the non-degenerated case without any repeated eigenvalues. After perturbation, the eigenvalue λ_k is adjusted to $\lambda_k+\Delta\lambda_k$, and the corresponding eigenvector is adjusted to $x_k+\Delta x$. By multiplying the eigenfunction, we have

$$(A^R+\Delta A)(x_k+\Delta x_k)=(\lambda_k+\Delta\lambda_k)(x_k+\Delta x_k) . \tag{3}$$

By x_k^T and neglecting the second-order terms $x_k^T\Delta A\Delta x_k$ and $\Delta\lambda_k x_k^T\Delta x_k$, we obtain

$$\Delta\lambda_k \approx \frac{x_k^T\Delta A x_k}{x_k^T x_k} . \tag{4}$$

Using the perturbed eigenvalues while keeping eigenvectors unchanged, the perturbed matrix can be obtained,

$$A'=\sum_{k=1}^{N}(\lambda_k+\Delta\lambda_k)x_k x_k^T , \tag{5}$$

which can be considered a linear approximation of the given network A if the expansion is based on A^R.

Next, considering the adjacency matrix contains repeated eigenvalues. If λ_{ki} is eigenvalues, the index i denotes M related eigenvectors of the same eigenvalues and the index k denotes different eigenvalues. It is given that any linear combination of eigenvectors belonging to the same eigenvalue is still an eigenvector. After adding a perturbation into the network, we choose the degenerate eigenvalues, which can be changed successively into the perturbed nondegenerate eigenvalues. If we define the chosen eigenvectors to be $x'_{ki}=\sum_{j=1}^{M}\beta_{kj}x_{kj}$, the eigenfunction becomes

$$(A^R+\Delta A)x'_{ki}=(\lambda_{ki}+\Delta\lambda'_{ki})x'_{ki} , \tag{6}$$

giving us

$$\Delta\lambda'_{ki}\sum_{j=1}^{M}\beta_{kj}x_{kj}=\sum_{j=1}^{M}\beta_{kj}\Delta A x_{kj} , \tag{7}$$

for any $n=1\cdots M$, left multiplying Equation (7) by x_{kn}^T, we obtain

$$\Delta\lambda'_{ki}\beta_{kn} = \sum_{j=1}^{M} \beta_{kj} x_{kn}^{T} \Delta A x_{kj} \ . \tag{8}$$

Written in matrix form, Equation (8) becomes

$$WB_k = \Delta\lambda'_k B_k \,, \tag{9}$$

where $W_{ni} = x_{kn}^{T}\Delta A x_{ki}$, which is a $M \times M$ matrix, and B_k is the column vector of β_{ki}. Then, according to eigenfunction (9), we obtain $\Delta\lambda'_k$ and B_k; the perturbed adjacency matrix A' is obtained by simply replacing x_k and $\Delta\lambda_k$ in Equation (5) with x'_k and $\Delta\lambda'_k$.

Matrix eigenvectors can reflect the network structural features. If the eigenvectors of matrix A' and matrix A are nearly the same, it indicates that the perturbation set does not significantly change the structural features. In other words, the network is of high structural consistency. All unobserved links (including perturbed links) are ranked in descending order according to their corresponding scores in perturbed matrix A'. Denote the set of top-L links as E^L, where $L = |\Delta E|$. Structural consistency σ_c is defined as the ratio of shared links between ΔE and E^L to L, follow as

$$\sigma_c = \frac{|E^L \cap \Delta E|}{L} . \tag{10}$$

Figure S1 shows how to calculate the structural consistency σ_c of a toy example. The left figure shows the adjacency matrix A, where the number in each square is the corresponding value of the matrix element. The second figure represents the matrix A^R, which is obtained by randomly removing a fraction of the observed links. The removed links, namely, $(1,4)$, $(3,8)$, $(5,10)$, $(7,8)$, $(7,10)$ and $(9,10)$, constitute the perturbation set ΔE. Obviously, $L = |\Delta E| = 6$. The right figure is the perturbed matrix A'. By ranking the unobserved links in A^R according to their corresponding values in A', we obtain the top-L links in E^L as $(1,4)$, $(6,8)$, $(3,8)$, $(5,10)$, $(3,9)$ and $(7,8)$. Therefore, there are four shared links between ΔE and E^L, $\sigma_c = 4/6 \approx 0.67$.

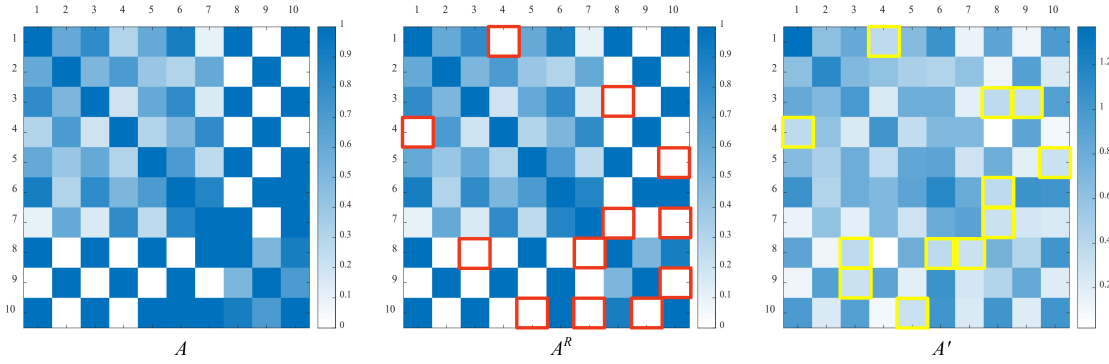

Figure S1. Toy example of σ_c calculation.

2 Structural perturbation method for lncRNA–miRNA interaction prediction

Generally, the link prediction problem of a network is how to estimate the probability of the existence of unobserved links according to known topological information. The network structure perturbation involved in the structure consistency calculation process can be used to predict the missing links (Lü et al., 2015).

For the lncRNA–miRNA bilayer network A, taking 5-fold cross-validation as an example, the observed links in the original adjacency matrix ($LMnet$) are randomly divided into 5 equal sized subsets. Of these 5 subsets, one is selected as the probe set, and the others, together with $LSnet$ and $MSnet$, as the training set. Next, we randomly remove a fraction of links from training sets to constitute the

perturbation set. The perturbation matrix can be calculated as A', see the section 1 for details. The final prediction matrix $\hat{A}'$ is obtained by averaging over ***t*** independent selections of the perturbation set. In this way, the elements in prediction matrix $\hat{A}'$ can be regarded as a score between a pair of nodes of the bilayer network A. The scores in $\hat{A}'$ determine the extent of all unobserved lncRNA–miRNA interactions, and we assume that the higher the score, the more likely the potential interaction will be.

3 References

Lü L, Pan L, Zhou T, Zhang YC, and Stanley HE. 2015. Toward link predictability of complex networks. Proc Natl Acad Sci U S A 112:2325-2330. 10.1073/pnas.1424644112

Zeng X, Liu L, Lu L, and Zou Q. 2018. Prediction of potential disease-associated microRNAs using structural perturbation method. Bioinformatics 34:2425-2432. 10.1093/bioinformatics/bty112
